# The inhibitory effect of Mesembryanthemum edule (L.) bolus essential oil on some pathogenic fungal isolates

**DOI:** 10.1186/1472-6882-14-168

**Published:** 2014-05-23

**Authors:** Beauty E Omoruyi, Anthony J Afolayan, Graeme Bradley

**Affiliations:** 1 Department of Biochemistry and Microbiology, University of Fort Hare, Private Bag X1314, Alice 5700, South Africa; 2 Department of Botany, University of Fort Hare, Private Bag X1314, Alice 5700, South Africa

**Keywords:** *Mesembryanthemum edule*, Essential oil, GC/MS, Antifungal activity, Opportunistic fungi

## Abstract

**Background:**

*Mesembryanthemum edule* is a medicinal plant which has been indicated by Xhosa traditional healers in the treatment HIV associated diseases such as tuberculosis, dysentery, diabetic mellitus, laryngitis, mouth infections, ringworm eczema and vaginal infections. The investigation of the essential oil of this plant could help to verify the rationale behind the use of the plant as a cure for these illnesses.

**Methods:**

The essential oil from *M. edule* was analysed by GC/MS. Concentration ranging from 0.005 - 5 mg/ml of the hydro-distilled essential oil was tested against some fungal strains, using micro-dilution method. The plant minimum inhibitory activity on the fungal strains was determined.

**Result:**

GC/MS analysis of the essential oil resulted in the identification of 28 compounds representing 99.99% of the total essential oil. A total amount of 10.6 and 36.61% constituents were obtained as monoterpenes and oxygenated monoterpenes. The amount of sesquiterpene hydrocarbons (3.58%) was low compared to the oxygenated sesquiterpenes with pick area of 9.28%. Total oil content of diterpenes and oxygenated diterpenes detected from the essential oil were 1.43% and 19.24%. The fatty acids and their methyl esters content present in the essential oil extract were found to be 19.25%. Antifungal activity of the essential oil extract tested against the pathogenic fungal, inhibited *C. albican*, *C. krusei*, *C. rugosa*, *C. glabrata* and *C. neoformans* with MICs range of 0.02-0.31 mg/ml. the activity of the essential oil was found competing with nystatin and amphotericin B used as control.

**Conclusion:**

Having accounted the profile chemical constituent found in *M. edule* oil and its important antifungal properties, we consider that its essential oil might be useful in pharmaceutical and food industry as natural antibiotic and food preservative.

## Background

The global epidemics of HIV/AIDS appear to have stabilized in most regions. However, Sub-Saharan Africa remains heavily affected region according to the report of UNAIDS [1]. Among the Sub-Saharan Africa countries, South Africa happened to be the largest burden of HIV/AIDS worldwide with an estimate of 5, 38 million out of 50.6 million indigenes in 2011 [2]. Majority of people living with HIV/AIDS are vulnerable to developing fungal illness because of their suppressed immune systems [3,4]. Fungal infections remain a significant cause of gastrointestinal disease, a consequence of HIV/AIDS contaminants especially in immunocompromised individuals [3,4]. The incidence of re-occurring fungal associated HIV/AIDS has increased dramatically. *Candida albicans* is one of the major causes of mucosal and bloodstream infections with over 85% to 95% cases reported if not treated [4]. *Cryptococcus neoformans*, a facultative organism that is very unique in attacking the lymphocytic cells, thereby creating a major gate way to HIV target. Meningitis, including lung infections are the common diseases related to *C. neoformans*[5].

*Candida glabrata* currently ranks the second or third causative agent of oral, vaginal, or urinary infections, which is often known as nosocomial disease. Its resistant mortality rates in compromised patients are very difficult to treat, especially with fluconazole drug [6]. Susceptibility of population with suppressed immunological defences against *Candida rugosa* infection in HIV/AIDS has emerged in spreading bovine mastitis in trauma patients [7,8]. Overall, *Candida krusei* ranked the fifth most common species that tends to be relatively seen in immunocompromised patients [8].

Over the years, the prevalence of fungal infection and its resistance to antibiotics drugs has brought to knowledge the importance to search for alternative treatments against infections [9]. It is noteworthy that researchers have directed their attention towards medicinal plants to develop better drugs against fungal infections. Traditional medicines have played an important role in health services around the globe, especially in South Africa due to wide arrays of phytochemicals with therapeutic properties [10]. Naturally, plants possesses free radical scavenging molecules, such as vitamins, terpenoids, phenolic acids, tannins, flavonoids, alkaloids, and other metabolites, which are rich in antioxidant with antimicrobial properties [11,12]. The ingestion of these natural antioxidants has shown to enhance the immune defence, reduce risks of cancer, cardiovascular disease, diabetes, and other diseases associated with ageing [12,13]. Owing to this fact, majority of South African population relies heavily on the use of plants and plant extracts for their well beings. Hence, much attention has been drawn to plant-derived fungicides in recent years for the replacement of modern drugs [14]. Essential oil and their volatile constituents derived from medicinal plants have been reported to possess potent antifungal activities [15]. Majority of individuals who use essential oils from plant is less likely to contract infections disease [16]. Moreover, oils users who eventually contract an infectious disease trend to recover faster than those using antibiotics [17].

In South Africa, essential oils are usually used to preserve food against the growth of organisms. Thus many of these essential oils from medicinal plants are cheaply distributed and sold in the local market centers due to increased demands [2]. The high reliance on medicinal plants for health purposes necessitates the scientific validation of their therapeutic value and safety.

*Mesembryanthemum edule* (L.) bolus is an edible growing ground-cover plant commonly found in the costal districts of Eastern Cape of South Africa. The Xhosa-speaking people in this province usually administered alcohol, aqueous and essential oil extracts for the management of diseases common with HIV/AIDS infection [18]. Based on the ethnomedical information on this plant, the crude essential oil extract was screened for activity against *Candida albican*, *C. rogusa*, *C. krusei*, *C. glabrata* and *C. neoformans*. The activities of *M. edule* on mycobacteria causing tuberculosis (TB) have been described [14], but reports on the biological effect of its essential oil on pathogenic fungal isolated from HIV/AIDS patients are limited. The aim of this research is to evaluate the inhibitory potential of *M. edule* essential oil against fungal isolated from HIV/AIDS patients. This study may justify its authentication to be used as complementary and alternative medicines.

## Methods

### Plant material

After obtaining the human ethics certificate (BRA0S1OMUO1) approved by the University of Fort Hare’s research ethics committee, the survey of this medicinal plant was carried out in June 2012, fresh leaves of *M. edule* were supplied by herbalist from Nkonkobe Municipality. The taxonomical identity of the plant was confirmed by a botanist Prof. DS Grierson and a voucher specimen was kept in the Griffin Herbarium of the Botany Department, University of Fort Hare as (Omo 2011/1-Omo 2011/19) [18].

### Essential oil

Volatile oil from the fresh leaves (500 g) was extracted for 3 h using a hydro-distiller (Clevenger’s-type apparatus) in a 5-L round bottom flask fitted in a condenser. This process of extraction was repeated by another 500 g of the fresh leaves.

### Gas chromatography–mass spectroscopy analysis

The essential oil extract was subjected to GC-MS analysis for identification of components in the department of Botany, University of Forth Hare. This was carried out using GC-MS (HP 6890) with a mass selective detector (HP5973). Identification of the components of essential oils was accomplished by comparison with the standards available in the database. The quantity of compounds was calculated by integrating the peak areas of spectrograms. A needle with the sample material (essential oils tested) was inserted directly into the inlet of a Hewlett Packard (HP 6890, USA) Gas Chromatograph. The temperature of the injection port was maintained at 220°C while the pressure at the inlet was maintained at 3.96 psi. A HP-5 MS (cross-linked 5% Phenyl Methyl Siloxane) column (30 m × 0.25 mm × 0.25 μm film thickness) was temperature- programmed from 60 to 150°C at 3°C min-1 after a 3 min delay. Helium was used as a carrier gas at 0.7 ml min-1. Mass spectra were recorded by a 5973 series Mass Selective Detector (MSD) [19].

### Calculation of oil yield

Prior to the final extraction and obtaining the oil, a clean bottle of known mass was made available. At the end of extraction process, the essential oil obtained was carefully transferred into the bottle and the final mass noted. The yield was obtained as follows: Mass of plant material distilled (g) = X; Mass of empty bottle (g) = A; Mass of bottle + oil extracted (g) = B; Mass of oil (g) = (B – A); Percentage (%) yield = [(B-A) ÷ X] 100 (Table 1). The essential oil was diluted in methanol (20% v/v) and a working concentration ranging between 0.005-5-mg/ml was used for the determination of Minimum Inhibitory Concentration (MIC).

**Table 1:** **Percentage yield essential oil from *M. edule *leaves**

**Item**	**Essential oil of leaf**
Percentage yield	4.21%
Colour	A very pale yellow
Solubility in methanol 20%	1 in 1/V

### Microorganisms and growth media

The fungi used in this study were chosen primarily on the basis of their importance as common pathogens of human infected with HIV/AIDS. Strains from the American type culture collection (ATCC) were used, including *C. albicans* ATCC 2091, *C. krusei* ATCC 204305, *C. glabrata* ATCC 2001, *C. rugosa* ATCC 10571 and *Cryptococcus neoformans* ATCC 66031. Both Sabouraud dextrose agar (SDA) and Sabouraud dextrose broth (SDB) were prepared according to the manufacturer’s instructions. Each fungus was grown for 48 hour at 28°C in Sabouraud Dextrose Agar (Merck) plates. Scrape cell mass were transferred from each solid culture to 3 ml saline solution and then adjusted to 0.5 Mc Farland standard, which was confirmed by spectrophotometric reading at 580 nm [20]. Cell suspensions were finally diluted to 10^4^ CFU/ml for the use in the assays.

### Minimum Inhibitory Concentration (MIC)

The micro-dilution method using Sabouraud dextrose broth was employed to determine the minimum inhibitory concentration (MIC) of the plant extracts using 96 well microtitre plates. Firstly, an initially, 120 μl of sterile distilled water was added into each well of the first (A) and last (H) rows and also into all the wells of the last column (12). Secondly, 120 μl of SDB was added into each well of the second row (B) and 150 μl of same SDB was added into the remaining wells of the first column and then a 100 μl into the rest of the wells from the second column rightward. Fifty microlitre of the essential oil was then added into the third well of the first column, while 50 μl of the positive and negative control were separately added into the remaining wells of the first column. Following two-fold serial dilution method, each contents from the first column (starting from the third row) was mixed by transferring 100 μl into the second well of the same row and the procedure was repeated up to the 11th well of the same row and the last 100 μl from the 11th well was discarded. Hence various concentrations of the diluted essential oil ranging from 5 mg/ml to 0.005 mg/ml were prepared in the wells, following the two-fold dilution method. Thereafter, 20 μl of 0.5 Mc- Farland fungal suspensions was inoculated into the wells except those which contained sterile distilled water. Each treatment was performed in triplicates. The growth of the fungi was measured by determining the absorbance at 620 nm with a microtitre plate reader before and after incubation. Plates were incubated at 37°C for 24 hours. The lowest concentration which inhibited the growth of the fungi was considered as the minimum inhibitory concentration (MIC) of each extracts.

### Statistical analysis

The antifungal experiments were made in triplicates and the data is reported as mean ± SD for (n = 1x3). Analysis of variance was performed by one way ANOVA using software statistical 5.5 (Stat Soft Inc, Tulsa, Ok). A probability value at P <0.05 was considered statistically significant.

## Results and discussion

### Percentage chemical compounds of the essential oil

**Figure 1:**
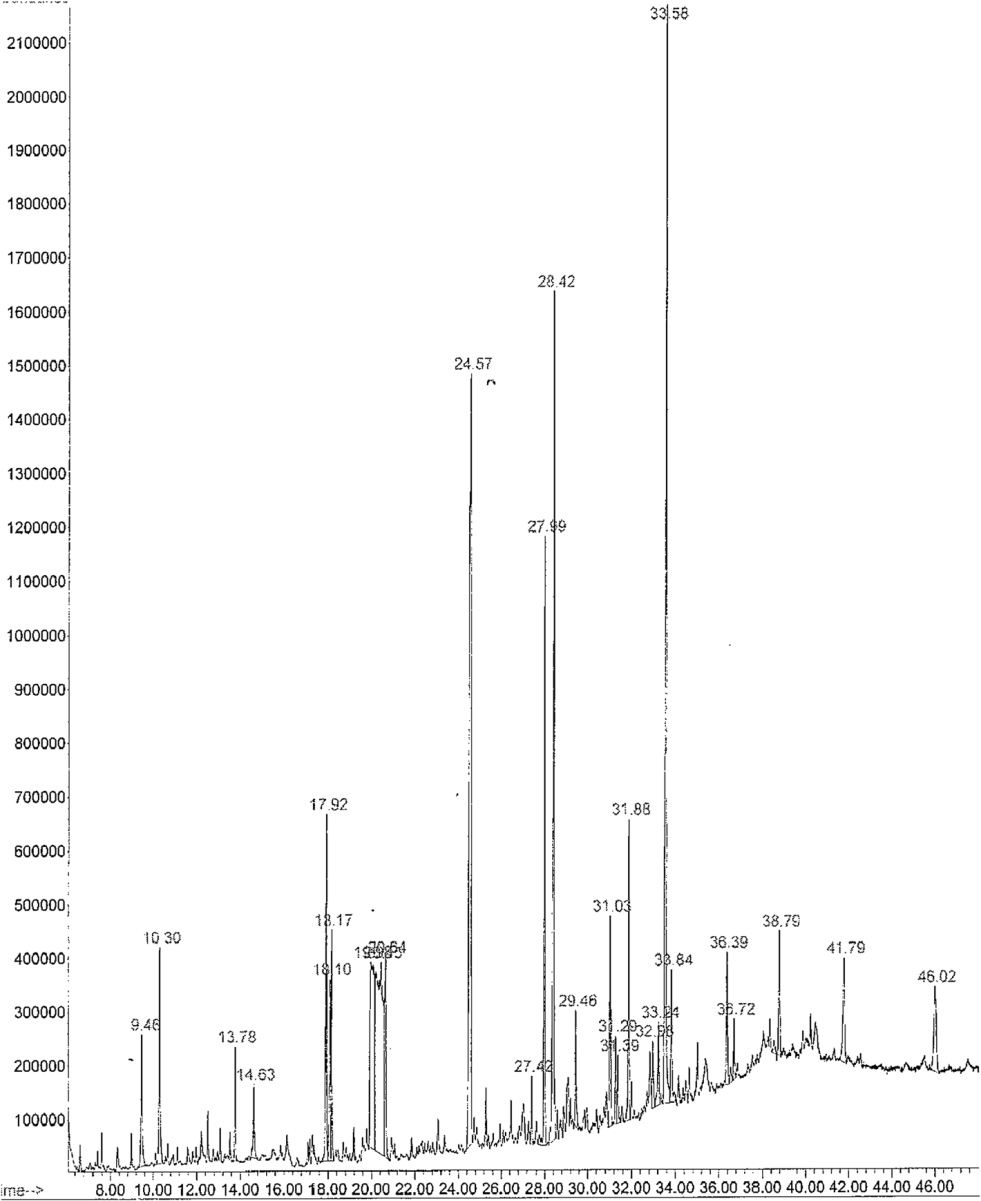
**A typical Gas chromatography profile showing the chemical analysis of *M. edule *** **essential oil.**

Hydro-distilled essential oil from fresh *M. edule* leaves analyzed by GC-MS resulted in the identification of 28 compounds representing 99.99% of the total essential oil. The essential oil was pale yellowish liquid with a fine-agreeable characteristic aroma. The major compounds of the essential oil found based on their mass spectra peaks (Figure 1) were the Tetra-decamethylcyclo-heptasiloxane with area peak of 23.81%, followed by Tetra-cosamethylcyclo-dodecasiloxanes (22.51%), Octadecane (2.56%), Nephthalene (3.93%) and Eicosane (4.0%), Table 2.

**Table 2:** **Compounds obtained from GC/MS analysis of *M. edule *leaf part essential oil**

**Percentage composition of *M. edule *essential oil analysed by GC/MS**				
**PK/No**	**Compounds**	**Kovats index**	**Peak %**	**Chemical formula**
	***Monoterpenes***		**10.6**	
1	Isoterpinolene	1429	0.95	C_10_ H_16_
2	Nephthalene, 1,2-dihydro-2,5,8-tri	1546	2.03	C_12_H_10_
3	Nephthalene, 1,2-dihydro-2,5,8-tri	1548	1.90	C_12_H_10_
4	Bistrimethylsilyl N-acetyl EICOSAS	1978	5.72	C_15_H_33_NO_5_Si_3_
	***Oxygenated monoterpenes***		**36.61**	
5	Mercaptoacetic acid, bis (trismethylsilyl)	3740	2.07	C_8_H_20_O_2_SSi_2_
6	Eicosamethylcyclodecasiloxane	1936	2.58	C_8_H_24_O_4_Si_4_
7	N-Octanol	1297	1.59	C_8_H_18_O
8	Nonylaldehyde	1345	2.29	C_9_H_18_O
9	Trans-β-demascenone	1538	3.42	C_13_H_18_ O
10	Trans-2-tridecenal	1406	0.85	C_13_H_24_O
11	Tetradecamethylcycloheptasiloxane	1627	7.39	C_14_H_42_O_7_Si_7_
12	Tetradecamethylcycloheptasiloxane	1646	13.57	C_14_H_42_O_7_Si_7_
13	Tetradecamethylcycloheptasiloxane	1654	2.85	C_14_H_42_O_7_Si_7_
	***Sesquiterpenes***		**3.58**	
14	Octadecane	1991	0.64	C_18_H_38_
15	Octadecane	2092	1.12	C_18_H _38_
16	1-octadecene	2266	0.80	C_18_H_36_
17	Nonadecane	2284	1.02	C_18_H_40_
	***Oxygenated sesquiterpene***		**9.28**	
18	2-pentadecanone,6,10,14-trimethyl	2014	9.28	C_18_H_36_O
	***Diterpenes***		**1.43**	
19	Eicosane	2215	0.65	C_20_H_42_
20	Eicosane	2439	0.78	C_20_H_42_
	***Oxygenated diterpenes***		**19.24**	
21	Phytol (2-Hexadecen-1-o1, 3,7,11,15-tetramethyl)	2289	12.41	C_20_H_40_O
22	Trisiloxane,1,1,1,5,5,5-hexamethyl-3-[(trimethylsilyl)oxy] (Tetracosamethylcyclododecasiloxane)	2302	1.64	C_24_H_72_O_12_Si1_2_
23	Tetrasiloxane,1,1,1,5,7,7,7-heptamethyl-3, bis[(trimethylsilyl)oxy] (Tetracosamethylcyclododecasiloxane)	2420	1.66	2 C_24_H_72_O_12_Si1_2_
24	3-Isopropoxy-1,1,1,7,7,7-hexamethyl-3,5,5-tri(trismethylsiloxy) tetrasiloxane (Tetracosamethylcyclododecasiloxane)	2538	1.69	2 C_24_H_72_O_12_Si1_2_
25	Tetrasiloxane-1,1,1,5,7,7,7-heptamethyl-3,3 bis[(trismethylsilyl)oxy)] (Tetracosamethylcyclododecasiloxane)	2680	1.84	2 C_24_H_72_O_12_Si1_2_
	***Fatty acids***		**19.25**	
26	Benzoic acid, 2,5-bis (trimethylsiloxy-,trimethylsilyl ester (Tetracosamethylcyclododecasiloxane)	1841	15.68	C_16_H_30_O_4_ Si_3_
27	Hexadecanoic acid, ethyl ester	2183	0.89	C_18_H_36_O_2_
28	Hexadecanoic acid, 1-methylethyle ester	2215	2.68	C_19_H_38_O_2_
	Total compounds (%)		**99.99**	

Bold data **(10.6; 36.61; 3.58; 9.28; 1.43; 19.24; 19.25; 99.99)** represents the percentage peak area values calculated for each terpenes and fatty acids hydrocarbon.

The use of some of these chemical compounds has been well studied. Compounds such as Tetra-cosamethylcyclo-dodecasiloxanes and Tetra-decamethylcyclo-heptasiloxane that ends with ‘siloxanes’ belongs to the wider class of organosilicon [21]. These compounds are made up of both organic and inorganic chemical compounds comprised of silicon, oxygen, carbon and hydrogen [22]. Siloxanes are commonly used in the cosmetic industries to produce deodorants, sunblocks, hairsprays and skincare [21]. In addition, siloxanes are an important product in the cook ware industry and kitchen utensils [21]. They are also used as effective industrial cleaning agents and in dry cleaning industries. In terms of properties, siloxanes are a good source of electric insulation, low chemical reactivity, low toxicity, high gas permeability, excellent resistance to oxygen, zone and UV light. Naphthalene is another chemical compounds derived from crude oil. It is a bicyclic aromatic hydrocarbon that is use as insecticide and as a repellent [23].

Majority of the volatile components analysed from plant essential oil largely belong to terpene. Terpenes are known to have strong biological activities and they are in involved in plant defences [24]. It has been well documented that the intake of terpenes can reduce accumulated toxins from the liver and kidneys in the body system [25]. In this study, Isoterpinolene (0.95%), Nephthalene (3.93%) and Bistrimethulesilyl N-acetyl (5.72%), were identified as monoterpenes respectively. Oxygenated monoterpene were found to be the highest (36.61%) constituents in the *M. edule* essential oil. Over the years, essential oil containing monoterpene hydrocarbons has offered a variety of healing properties, especially their ability to restore correct information in the DNA of a living cell and enhancement of other therapeutic components [26,27]. Isoterpinolene, one of the major monoterpenes observed in the study has been found capable of protecting human cells from free radical mediate oxidative stress [28]. It has been said that the oxygenated monoterpene compounds are more valuable than the monoterpene hydrocarbons due to their contribution to the fragrance of the essential oil [29].

Octadecanes (1.76%), 1-octadecane (0.80%) and Nonadecane (1.02%) were observed as sesquiterpene hydrocarbons in the *M. edule* essential oil. Essential oil containing sesquiterpenes have been used as therapeutic effect against inflammatory and allergic infections [30,31]. Research has found that people who consistently use sesquiterpenes essential oil have a higher level of resistance to illness than the average person [32]. Further indications revealed that if such individual eventually falls ill, he or she has a tendency of recovering 60–70% faster than those who do not use essential oils [32,33].

Two Eicosane with a total area peaks of 1.43% were the major concentrated diterpenes detected from the *M. edule* essential oil. Oxygenated diterpene constituents accounted the third most concentrated hydrocarbons found in leaves, with a total essential oil content of 19.24%. Of these, Phytol content gave the highest amount with area peak of 12.41%, followed by all the Tetra-decamethylcyclo-heptasiloxanes, having area peak of 6.83%. Total amount of fatty acids and their methyl esters content present in the essential oil extract were found to be 19.25%. From Table 2 it is clear that benzoic acid represent the highest amount of 15.68% fatty acids of the essential oil.

Several bioactive compounds have been isolated from *M. edule*, such as phenolics, flavonoids, proanthocyanidins, alkaloids, saponins, tannin, rutin, cactichin, ferulic acid hyperoside, oleanolic acid, catechin and epicatechin [34-36]. Unfortunately, there is no available information on the profile chemical constituents from *M. edule* leaf essential oil. The different phytochemical constituents of monoterpene, sesquiterpenes, diterpenes and fatty acid esters have been reported to have antioxidant, antimicrobial, immune-modulating activities [37-39].

Microbiological screening of essential oil from many plants (*Pimpinella anisum* L. (anised), *Syzygium aromaticum* L. (clove), *Cuminum cyminum* L. (cumin), *Allium sativum* L. (garlic), *Laurus nobilis* L. (laurel), *Citrus sinensis* (L.) Osbeck (orange sweet), and *Origanum vulgare* L. (oregano), *Tulbaghia violacea* Harv L.F and *Eucalyptus grandis* W.Hill ex Maiden) have earlier been studied to have high antibacterial, antifungal, antiviral, antiparasitic and antidermatophytic properties [40-42]. From the antifungal results presented in Table 3, the MIC of the essential oil effectively inhibited the growth of the organisms when compared to nystatin and amphotericin B used as control. The extent of inhibition on the fungal growth is dependent on the concentration used. *Candida albican* and *C. krusei* which are the most dangerous organisms in human system had the most sensitive treatment of 0.02 and 0.04 mg/ml activity. These findings agree with studies done on the *Candida* strain isolated from infants and standard strains [43]. Both strains were greatly inhibited by 80.95% and 14.23% essential oil of thyme, pennyroyal and lemon [43].

**Table 3:** Minimum inhibitory concentration (MIC) of the extracts against the five fungal

**Minimum inhibitory concentration**			
**Test organisms**	**Essential oil (mg/ml)**	**Nystatin (mg/ml)**	**Amphotericin B (mg/ml)**
*C. albican*	0.02	0.02	0.02
*C. krusei*	0.04^a^	0.02^b^	0.009^c^
*C. rugosa*	0.08^a^	0.02^b^	0.009^c^
*C. glabrata*	0.31^a^	0.04^b^	0.02^b^
*C. neoformans*	0.08^a^	0.009^b^	0.02^c^

^abc^Values within rows are significantly different (*p* > 0.05). The minus sign (−) indicate negative result.

Minimum inhibitory activity of the essential oil against *C. rugosa* and *C. neoformans* (0.08 mg/ml) was significantly different from that of *C. glabrata* at 0.31 mg/ml. Antifungal activity of *Lavandula viridis* L. essential oil against *Cryptococcus neoformans* was 0.64 μl/ml, which is significantly higher than our result [44]. Other observations from Saeid and Seddighe [43], reported 2.3% activity of essential oil against *C. glabrata.*

## Conclusion

Conclusively, the results obtained from the GC-MS resulted in the identification of 28 hydrocarbons of the total essential oil. The phytoconstituent present in the essential oil are in the family of monoterpenes, sesquiterpenes, diterpenes, and fatty acids esters. Oxygenated monoterpenes occupies the major constituents of the oil, followed by fatty acids and oxygenated diterpenes. The therapeutic potency of *M. edule* used as traditional medicine thus contains properties that inhibit the growth of fungi activity. The growth of *Candida albican* and *C. krusei* which are the most common agent in candiadiasis patients were greatly reduced by *M. edule* essential oil. *Mesembryanthemum edule* match a candidate species for future studies on novel and alternative remedy for the treatment of microorganism infections. However, further studies will be carried out to isolate and identify the active compounds, and to determine their exact mechanism of action.

## Abbreviations

HIV: Human immunodeficiency virus; AIDS: Acquired immunodeficiency syndrome; *M. edule*: *Mesembryanthemum edule*; UANOVA: Analysis of variance; MSD: Mass Selective Detector; GC-MS: Gas chromatography–mass spectrometry; ATCC: America type culture collection; MIC: Minimum inhibitory concentration.

## Competing interests

The authors declare that they have no competing interests.

## Authors’ contributions

BEO was responsible for the collection of plant materials from the traditional healers, carried out 90% of the experiments, and drafted the manuscript. GB edited the manuscript. AJA participated in the GC-MS analysis, supervised in the laboratory assay and made substantial contribution to revise the manuscript critically. All authors read and approved the final manuscript.

## Pre-publication history

The pre-publication history for this paper can be accessed here:

http://www.biomedcentral.com/1472-6882/14/168/prepub
